# Genome-wide association mapping and gene expression analysis identify *OsCPS1* as a new candidate gene controlling early seedling length in rice

**DOI:** 10.3389/fpls.2022.976669

**Published:** 2022-09-02

**Authors:** Yamei Ma, Jian Wang, Tifeng Yang, Jingfang Dong, Wu Yang, Luo Chen, Lian Zhou, Jiansong Chen, Bin Liu, Shaohong Zhang, David Edwards, Junliang Zhao

**Affiliations:** ^1^Rice Research Institute & Guangdong Key Laboratory of New Technology in Rice Breeding & Guangdong Rice Engineering Laboratory, Guangdong Academy of Agricultural Sciences, Guangzhou, China; ^2^School of Biological Sciences and Centre for Applied Bioinformatics, The University of Western Australia, Perth, WA, Australia

**Keywords:** rice, shoot length, genome-wide association study, *OsCPS1*, selection

## Abstract

High seedling vigor can improve the ability to compete against weeds and flooding at the seedling stage and is essential for the direct seeding of rice. Early shoot length is an important performance index in seedling vigor evaluation. However, information on the identity of rice germplasm with high seedling vigor, and the genetic basis of seedling vigor are not well understood. In this study, we have conducted a genome-wide association study using 302 international diverse rice accessions from the Rice Diversity Panel 2. Six quantitative trait loci (QTLs) were found to associate with shoot length (SL). The locus *qSL2* was further analyzed for candidate gene characterization. We identified *OsCPS1*, which encodes CDP synthase and functions in GA (Gibberellins) biosynthesis in rice, exhibits differential expression between long and short SL accessions. Using the *Nipponbare* genome sequence as the reference, we identified a 36 bp deletion in the 5’ UTR of *OsCPS1* in long SL accessions, which is absent in short SL accessions. GA content analysis showed that the levels of bioactive GA_1_ and GA_4_ are considerably higher in long SL accessions than in short SL accessions. Genome-wide gene expression analysis indicated the expression of some photosynthesis genes is higher in long SL accessions than in short SL accessions. In contrast, genes involved in ABA (Abscisic Acid)-activated signal pathway showed lower expression in long SL accessions. Population analysis across wild rice, *indica* and *japonica,* suggested that *OsCPS1* may be under selection in *japonica* during domestication. The results suggest that *OsCPS1* is a candidate gene underlying *qSL2*. These data provide a promising source for candidate genetic variation associated with seedling vigor, with practical applications in rice breeding.

## Introduction

Rice is one of the most important crops worldwide. Traditional rice cultivation systems involve the raising of nursery seedlings followed by transplanting the seedlings into paddy fields, requiring significant amounts of water, energy, and labor. Hence, an increasing number of rice farmers are adopting direct-seeding methods that have many advantages over traditional transplanting, including reducing water use, decreased labor, shorter crop duration, reduced methane gas emissions and lower costs (Abe et al., 2012; Mahender et al., 2015; Chakraborty et al., 2017). However, the lack of varieties suitable for direct seeding is a major constraint for large-scale implementation of direct seeding, since most modern cultivars are bred for transplanting and lack some of the traits that are important for direct seeding. Therefore, the breeding of elite varieties suitable for direct seeding is important for the future adoption of direct seeding practices in rice production.

Early seedling vigor (ESV) is one of the most important traits related to direct seeding. ESV reflects the ability of rapid and uniform germination, as well as seedling establishment (Ju et al., 2007). High ESV facilitates nutrient uptake to support shoot growth at the seedling stage, which is required for seedling establishment and weed competition (Rao et al., 2007; Anandan et al., 2016). The mining of important genes regulated ESV, and the breeding of rice varieties with high ESV would support an expansion of rice direct seeding production.

Early seedling vigor is a complex trait, mainly distinguished by the rate of germination and rapid early growth of both the shoot and roots. Several seedling vigor QTLs have previously been identified with biparental populations (Cheng et al., 2013; Zhang et al., 2017; Dimaano et al., 2020), recombinant inbred lines (Huang et al., 2004; Zhou et al., 2007; Xie et al., 2014; Singh et al., 2017) and association mapping studies (Dang et al., 2014; Wang et al., 2018). Seedling vigor QTLs have been detected using several different phenotypes, including germination index and germination rate (Wang et al., 2010), low-temperature vigor of germination (Najeeb et al., 2020), shoot length, root length, root number and weight of germinated seeds (Zhang et al., 2017; Wang et al., 2018; Dimaano et al., 2020). These studies have provided genetic insights into seedling vigor in rice. However, the identification of functional genes is rare, and the mechanism underlying rice early seedling vigor is largely unknown (Zhang et al., 2017).

Among all the traits determining ESV in rice, shoot length (SL) is one of the most important traits related to direct seeding. Rapid, uniform germination and vigorous seedling growth could contribute to early establishment and provide competitive advantage over weeds. Abe et al. (2012) identified QTLs for 14 days old seedling length, and demonstrated the height of seedlings in Dunghan Shali is controlled by a QTL with a major effect, *qPHS3-2*. Furthermore, they identified the gene *OsGA20ox1*, which is related to gibberellin (GA) biosynthesis, as a strong candidate gene for *qPHS3-2*. However, no further detailed results on functional genes or molecular mechanisms for SL have been reported.

In this study, we evaluated the shoot length (SL) phenotype using 302 international diverse rice accessions from the Rice Diversity Panel 2 (McCouch et al., 2016) at the early seedling stage. All accessions were sequenced to ~50X depth, and high-density SNPs were called for genome-wide association (GWAS) analysis. In total, 96 significant SNPs were identified that clustered into 6 regions, which were considered as QTLs, distributed on chromosomes 2, 4, 6, 11 and 12. Among these QTLs, the *qSL2* locus on chromosome 2 contributed 3.05% variation across in whole panel and 7.38% variation across the *indica* subpopulation. Based on gene functional annotation, gene expression and GA content analysis, the *OsCPS1* gene, which encodes an enzyme that participates in GA biosynthesis, was identified as the candidate gene for *qSL2*. Further analysis suggested a 36-bp deletion in the 5' UTR of *OsCPS1*, which strongly correlated with the expression difference of *OsCPS1*, may be the functional variation leading to the SL phenotype difference. Analysis of this variation and genomic sequence diversity surrounding *OsCPS1* across *indica* and *japonica* subpopulations revealed *OsCPS1* may be under selection in *japonica* during domestication. The identification of *qSL2* and the candidate gene *OsCPS1* provides a promising source of genetic variation for the molecular breeding of rice with high seedling vigor.

## Materials and methods

### Plant materials

A subset of Rice Diversity Panel 2 (RDP2; McCouch et al., 2016) consisting of 302 diverse accessions from 53 countries were used in this study. Seeds were provided by the International Rice Research Institute (IRRI).

### Evaluation of shoot length

Seeds for assessment were harvested at 30–40 days after flowering, air-dried under natural conditions and treated at 49°C for 3 days to eliminate dormancy. After sterilized with 3% sodium hypochlorite solution, the seeds were soaked in distilled water for 24 h, then placed in petri dish with wet filter paper and cultivated at 30°C under artificial illumination. After 5 days, the shoot lengths were measured manually. Each line had three replicate plates, each containing 30 seeds.

### GWAS

DNA was extracted from fresh leaf tissue using the CTAB (hexadecyltrimethylammonium bromide) protocol. DNA sequencing was performed on the Illumina NovaSeq6000 platform (BerryGenomics, China) and data aligned to the *Nipponbare* reference genome (MSU 7.0;^1^ Kawahara et al., 2013) using BWA (Li and Durbin, 2009). Nucleotide variants were called using GATK (Van der Auwera and O’Connor, 2020). SNPs were selected for GWAS analysis based on the criteria of missing data <15% and minor allele frequency of >0.05. GWAS was performed using a mixed linear model (MLM) with kinship matrix and principal component analysis in GAPIT 2 (Tang et al., 2016) and a cutoff threshold of –log10(*p*) = 4. Manhattan plots were produced using the R package ggplot2 (Wickham, 2016). Following GWAS analysis, significant SNPs within a 100 kb interval were classed as a locus, and candidate genes were identified from 100 kb upstream and downstream of the most significant SNP in each QTL.

### RNA-sequencing

Three long SL accessions and three short SL accessions were selected for RNA-sequencing based on haplotype analysis. Seeds were germinated in a petri dish in a growth chamber. RNA was extracted from five-day-old seedlings using the HiPure Plant RNA Mini Kit (Magen, Guangzhou, China) with three biological RNA replicates. RNA-Seq was conducted by Annoroad Gene Technology Co., Ltd. (Beijing, China). RNA samples were sequenced using an Illumina HiSeq-2,500, producing 10 Gb of raw sequencing data. Data analysis was conducted using HISAT2-Stringtie-Deseq2 pipeline (Pertea et al., 2016). Raw counts of each sample exported from Stringtie were imported and normalized by DEseq2 (Love et al., 2014). Genes with average read counts less than 10 in all samples were filtered out for further analysis. For each accession, transcript abundance was calculated as the mean normalized counts of three biological replicates. *p* values between long and short SL accessions were estimated by the Student’s *t*-test. The genes with corrected *p* values (false discovery rate or *q*-value) ≤0.05 were identified the differentially expressed genes. GO enrichment analysis was performed using DAVID (Sherman et al., 2022).

### Differential expression analysis of genes by qRT-PCR

First-strand cDNA was synthesized from 1 μg total RNA using the PrimeScript^TM^ RT reagent kit (Takara, Dalian, China). The house-keeping *ubiquitin* gene was used as an internal control. Real-time PCR was performed using the SYBR Premix ExTaq^TM^ kit (Takara, Dalian, China) and the Biorad CFX 96 Real-Time System (BioRad, Hercules, CA, United States). Relative expression levels were calculated with the 2^-△△CT^ method (Livak and Schmittgen, 2001).

### DNA diversity analysis

The sequences of *OsCPS1* of all 302 accessions and *rufipogon* was used for DNA diversity analysis. Sequences were imported into ClustalW to construct a nucleotide alignment matrix. Nucleotide diversity (π) values were calculated using VCFtools with 500 adjacent nonoverlapping windows and a 100-bp step (Danecek et al., 2011).

### Data availability statement

The RNAseq datasets are available at NCBI project number: PRJNA839180. The DNA sequencing datasets are available at NCBI project number: PRJNA820969. The wild rice sequencing data was downloaded from https://www.ncbi.nlm.nih.gov/, PRJNA657701.

### Data analysis

A phylogenetic tree was constructed by SNPhylo (Lee et al., 2014) using SNP data for all accessions. An LD heatmap was drawn with the R package LD heatmap. Student’s *t*-test was used to test the significance of difference.

### GA content

The GAs were extracted from five-day-old rice seedlings and assessed by HPLC-MS/MS. About 1 g of frozen rice sample was ground to a fine powder in liquid nitrogen, and 10 times the volume of acetonitrile solution was added followed by shaking at 4°C for overnight. The supernatant was extracted and the sample treated with another 5 times the volume of acetonitrile solution. The supernatants were combined and subjected to 35 mg solid-phase C18 extraction. Following centrifugation at 10000 rpm for 5 min, the supernatants was collected, dried under nitrogen gas, and redissolved in 400 μl of methanol. HPLC-MS/MS was then used to measure the GA content after passing through a 0.22-μm filter, using a 1,290 HPLC system (Agilent, Santa Clara, CA, United States) with a 6,500 Qtrap MS/MS (AB SCIEX, Framingham, MA, United States).

## Results

### Variation of shoot length in rice early seedling stage in an international diverse panel

**Figure 1:**
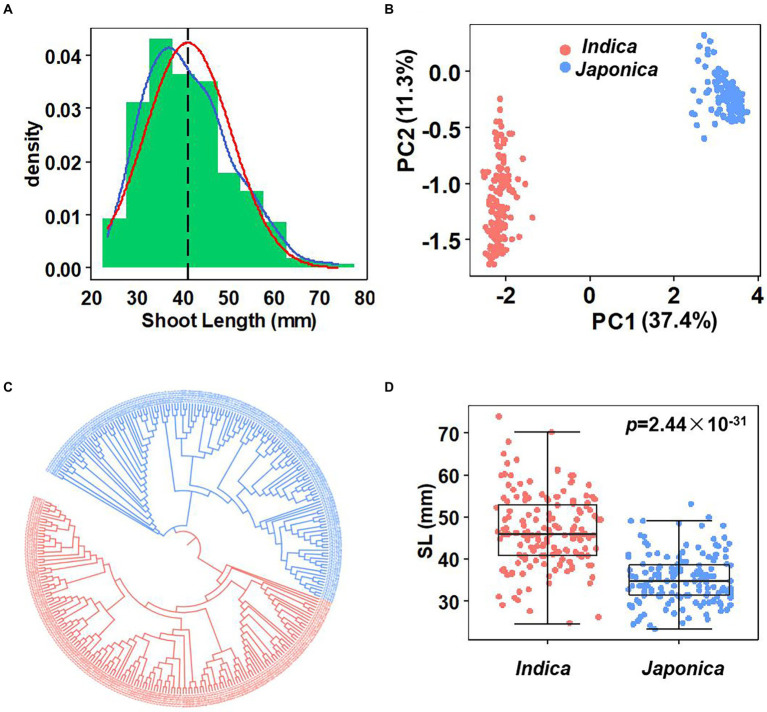
Phenotype variation and population structure. **(A)** Histogram of the SL. Blue line: Trendline, red line: Normal distribution line, black line: Mean of SL, mean = 41.02 (mm). **(B)** PCA of the population. **(C)** Phylogenetic tree of the population. The red and blue shape represent *indica* and *japoncia*, respectively. **(D)** SL variation in two subpopulations. Box edges represent the 0.25 quantile and 0.75 quantile with the median values shown by bold lines. Ymin lower whisker = smallest observation greater than or equal to lower hinge-1.5* IQR (interquartile range). Ymax upper whisker = largest observation less than or equal to upper hinge +1.5* IQR. Student’s *t*-test was used for statistical analysis.

A panel consisting of 302 diverse rice accessions was selected based on diversity and representativeness from an international rice panel (1,568 accessions; McCouch et al., 2016). Shoot length (SL) was measured and used for GWAS analysis. A wide variations in SL was observed, ranging from 23.3 to 74 mm, with an average of 41 mm (Figure 1A; Supplementary Table 1). The SL distribution was continuous and close to normal distribution. Particularly, two *indica* accessions, TI KU and MA GU ZI HE had an extreme SL phenotype, longer than 70 mm.

### Identification and mapping of QTLs for SL by GWAS

All 302 accessions were sequenced with the average depth of 50X. Sequencing data was mapped to the *Nipponbare* reference genome to call the SNPs. After removing loci with more than 15% missing data and minor allele frequency (MAF) less than 5%, we obtained 1,422,101 high-quality SNPs.

The population structure was evaluated by SNP phylogenetic and PCA analysis. The results showed that these accessions clearly clustered into two groups, representing 155 *indica* accessions and 147 *japonica* accessions (Figures 1B,C). Phenotype analysis indicated that *japonica* accessions had shorter shoot length than *indica* accessions (*p* < 0.001; Figure 1D). According to the LD decay analysis, the LD distance of the population used in this study is about 150–200 kb on chromosomes level (Supplementary Figure 1).

**Figure 2:**
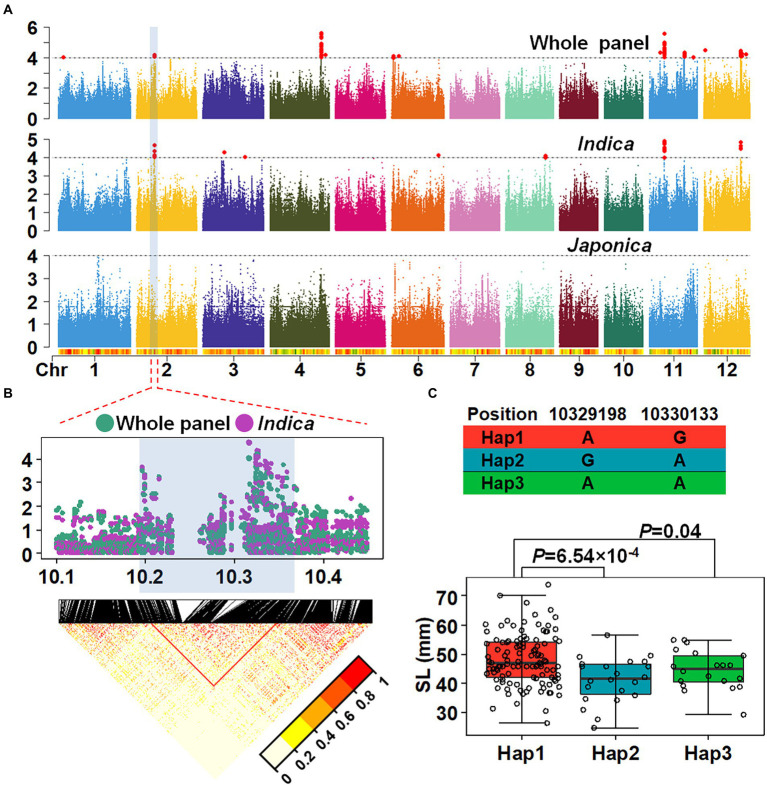
Identification and Mapping of QTL for SL through GWAS. **(A)** GWAS with shoot length with different population. **(B)** Local manhattan plots merged by the whole panel and *Indica* population. **(C)** Haplotype analysis with SL in *Indica* subpopulation. Student’s *t*-test was used for statistical analysis.

To determine genetic loci associated with SL, GWAS was performed using a mixed linear model (MLM). According to the LD decay results above, a region was considered as one QTL where it had more than two SNPs with −log10(*p*) > = 4 within a 200 kb window. The results of GWAS were shown using the Manhattan plot. Six QTLs were identified in the whole population (Figure 2A), distributed across chromosomes 2, 4, 6, 11, 12 and designated as *qSL2*, *qSL4*, *qSL6*, *qSL11-1*, *qSL11-2* and *qSL12* (Table 1). Among these QTLs, *qSL4* co-localized with a previously identified QTL for seedling vigor (Abe et al., 2012), demonstrating the reliability of the results. The remaining QTLs are newly identified. Due to the observed differences in SL between *indica* and *japonica*, GWAS was further conducted on the *japonica* and *indica* subpopulations, respectively. Four QTLs were identified in the *indica* subpopulation, while no QTL were identified in the *japonica* subpopulation (Figure 2A). Comparisons of the QTLs in different subpopulations demonstrated that *qSL2*, *qSL11-1* and *qSL12* can be identified in both the whole population and the *indica* subpopulation.

**Table 1:** QTLs for shoot length identified in the present study and their co-location QTLs identified in the previous studies.

Population	QTL	Chr	Peak position (bp)	*p*-value	MAF	Variation explained (%)	Co-location QTL/marker	Reference
Whole panel	*qSL2*	2	10,325,370	6.75E-05	0.372137	3.05%		
	*qSL4*	4	30,462,896	2.38E-06	0.351145	4.33%	*qSHL4*	Abe et al., 2012
	*qSL6*	6	860,755	7.85E-05	0.244275	2.99%		
	*qSL11-1*	11	8,933,830	2.76E-06	0.083969	4.27%		
	*qSL11-2*	11	21,052,417	4.58E-05	0.425573	3.20%		
	*qSL12*	12	22,087,359	3.62E-05	0.146947	3.29%		
*Indica*	*qSL_Ind_2*	2	10,316,768	2.12E-05	0.266026	7.38%		
	*qSL_Ind_8*	8	23,641,077	8.23E-05	0.105769	6.28%		
	*qSL_Ind_11*	11	8,933,830	1.23E-05	0.141026	7.83%		
	*qSL_Ind_12*	12	22,078,316	1.45E-05	0.141026	7.70%		

### Candidate gene analysis of *qSL2*

A QTL on chromosome 2 (*qSL2*) was further analyzed. The most significant SNP within *qSL2* interval was located at 10,325,370 bp on chromosome 2, explaining 3.05 and 7.38% of the phenotypic variation in the whole panel and *indica* subpopulation, respectively. According to the LD decay in the region, we delimited *qSL2* into a ~ 200 kb region (10.2–10.4 Mb on chromosome 2; Figure 2B; Supplementary Figure 1). Haplotype analysis identified three haplotypes based on the two most significant SNPs in the interval. Further analysis showed that 72.73% of the accessions harbor Hap1, 14.29% for Hap2 and 12.99% for Hap3 in the *indica* subpopulation (Supplementary Figure 2A). However, in the *japonica* subpopulation, 99.07% of the accessions harbored Hap1, 0% for Hap2 and 0.93% Hap3 (Supplementary Figure 2B). A similar result was obtained using SNP data from 3,000 rice accessions (Supplementary Figures 2C,D; Mansueto et al., 2017). Phenotypic variation related to haplotype was assessed and the results showed that in *indica* accessions with Hap1 showed longer SL than accessions with Hap2 (*p* value: 6.54 × 10^−4^) or Hap3 (*p* value: 0.04; Figure 2C).

**Figure 3:**
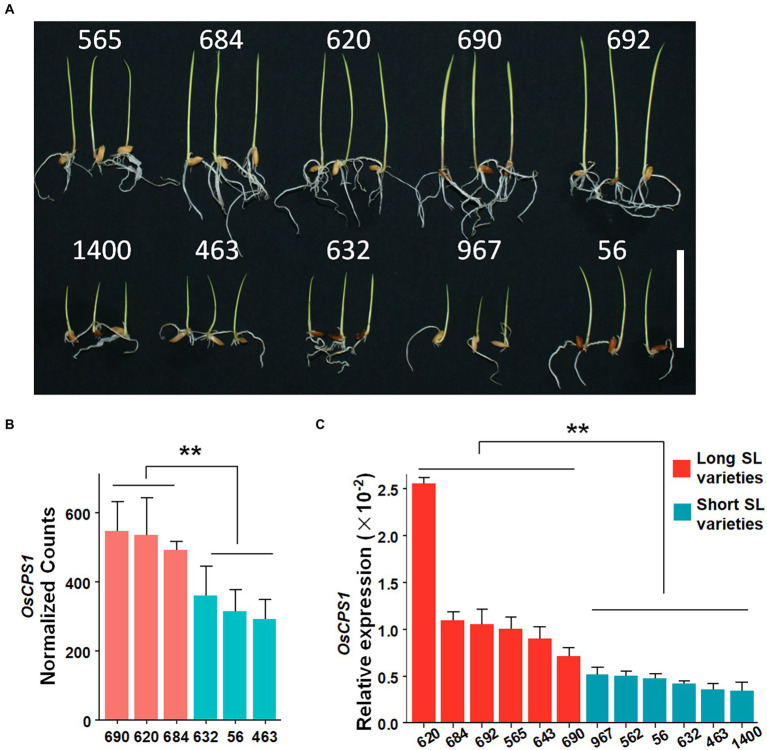
The SL phenotype and the candidate gene *OsCPS1* expression in different varieties. **(A)** The phenotype of representative varieties after 5 days germination. *Scale bar* 50 mm. **(B)** *OsCPS1* expression analysis by RNA-seq. Red bars represent long SL varieties, blue bars represent short SL varieties. Data are shown as means ± SD (*n* = 3). ^**^p < 0.01. *t*-test. **(C)** *OsCPS1* expression analysis by qRT-PCR. Red bars represent long SL varieties, blue bars represent short SL varieties. Data are shown as means ± SD. ^**^p < 0.01. *t*-test.

The *qSL2* genomic interval contains 24 annotated genes based on release 7 of the MSU Rice Annotation Project (Kawahara et al., 2013). To further assess these candidate genes, three long SL accessions (accession No. 690, 620 and 684) and three short SL accessions (accession No. 632, 56 and 463) were selected for whole genome expression analysis by RNA-seq (Figure 3A). 11 of 24 genes were expressed in shoot tissue, with LOC_Os02g17780 (*OsCPS1*) showing a significant difference (*p* < 0.01) in expression between long and short SL accessions (Figure 3B; Supplementary Figure 3). To further verify this result, we further performed qRT-PCR to determine the expression level of *OsCPS1* using six long SL accessions (accession NO. 690, 620, 684, 692, 565 and 643) and six short SL accessions (accession NO. 632, 56, 463, 967, 562 and 1,400). *OsCPS1* levels were significantly higher in long SL accessions than that in short SL accessions (Figure 3C). Previous studies have shown that *OsCPS1* encodes an ent-CDP synthase and functions in gibberellin biosynthesis (Otomo et al., 2004; Prisic et al., 2004; Sakamoto et al., 2004). Since GA is an important plant hormone that plays a central role in regulating growth and development, we hypothesized *OsCPS1* may be the functional gene of *qSL2*.

**Figure 4:**
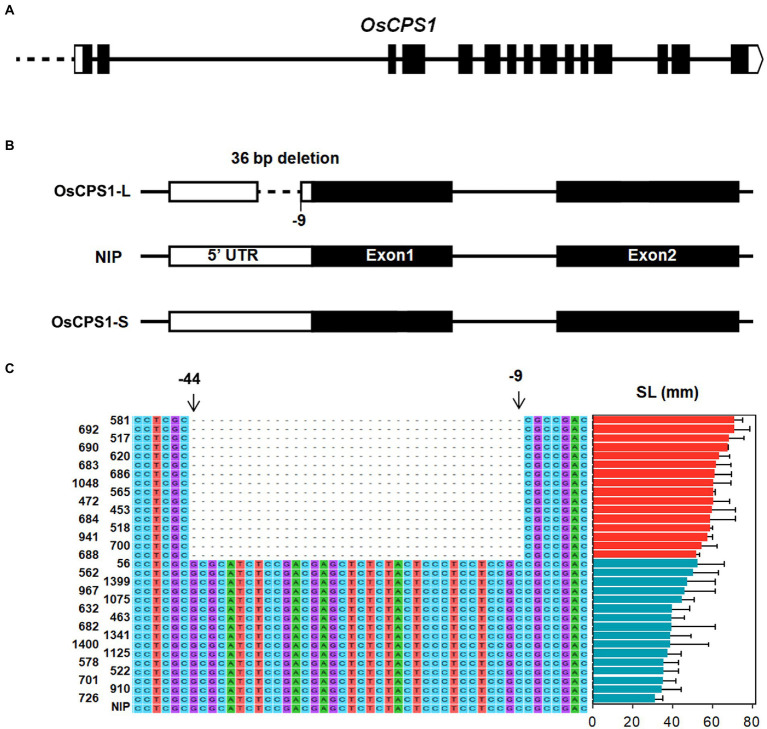
Sequence variations of *OsCPS1* in long and short SL varieties. **(A)** Gene structure of *OsCPS1*. Black rectangles and white rectangles indicate the exons, 3′ and 5′ UTR, respectively. **(B)** Indels in OsCPS1-L and OsCPS1-S comparing with Nipponbare (NIP). –9 represents the 9 bp upstream of start codon. **(C)** Sequence alignment with 36-bp Indel between long and short SL varieties. The sequence of *OsCPS1* in Nipponbare (NIP) was taken as reference. The sequences from −9 to −44 were deleted in long SL varieties.

To find the cause of differential expression of *OsCPS1* between long SL accessions and short SL accessions, we compared the sequence differences of *OsCPS1* between 16 long SL accessions and 16 short SL accessions, which were selected based on phenotype and haplotype analysis. As shown in Figures 4B,C, a 36 bp Indel in the 5' UTR that was located 9 bp upstream of the start codon of *OsCPS1* was found in long SL accessions (designed as OsCPS1-L), but not in short SL accessions (designed as OsCPS1-S). This sequence variation could be the cause for their differential expression in long and short SL accessions.

**Figure 5:**
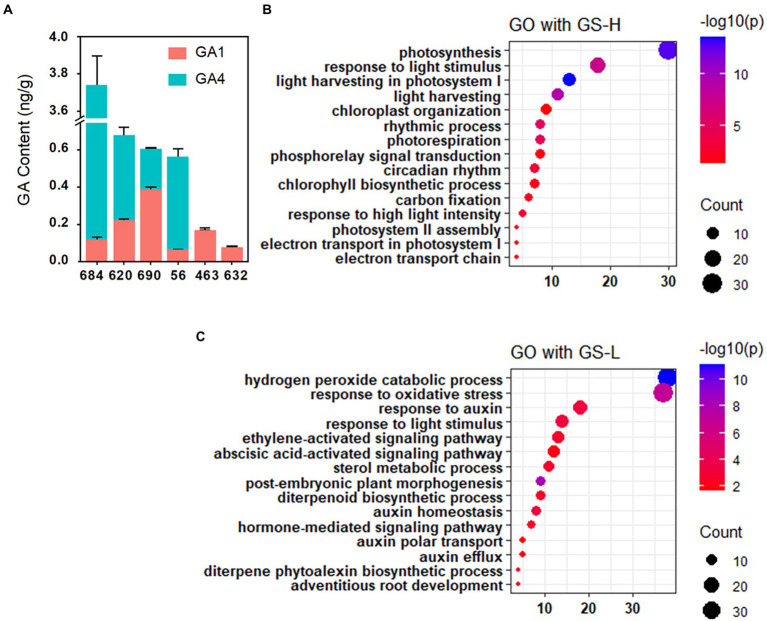
GA content analysis between long and short SL varieties and GO enrichment analysis. **(A)** GA_1_ and GA_4_ content analysis between long and short SL varieties. The results showing higher GA_1_ and GA_4_ levels in long SL varieties than short SL varieties. Data are shown as means ± SD (*n* = 3). **(B)** GO enrichment analysis with GS-H. GS-H: gene set with higher expression in long SL varieties than in short SL varieties. **(C)** GO enrichment analysis with GS-L. GS-L: gene set with lower expression in long SL varieties than in short SL varieties.

As *OsCPS1* is predicted to participate in GA synthesis, the GA content in shoot was determined using six rice accessions that were used for RNA-seq. The long SL accessions showed increased GA_1_ and GA_4_ level compared to the short SL accessions (Figure 5A). Since GA_1_ and GA_4_ are two major active GAs involved in regulating vegetative growth (Magome et al., 2013), these results further suggested that GA content may be affected by OsCPS1 and responsible for the SL phenotype.

To further confirm that *OsCPS1* is the candidate gene, we analyzed differentially expressed genes between long and short SL accessions by RNA-seq data. As shown in Figure 5B, genes with higher expression in long SL accessions than those in short SL accessions were significantly enriched for GO terms related to photosynthesis (GO:0015979), light harvesting (GO:0009765), chlorophyll biosynthetic process (GO:0015995) and carbon fixation (GO:0015977). In contrast, gene involved in abscisic acid-activated signaling pathway (GO:009738) showed lower expression in long SL accessions than in short SL accessions (Figure 5C). It has been reported photosynthesis-related genes can be induced by GA treatment, while ABA functions as an antagonist of GA (Gómez-Cadenas et al., 2001; Biemelt et al., 2004; Xie et al., 2016). The above results together suggested OsCPS1-mediated GA signaling may participate in regulating the SL phenotypes conferred by *qSL2*.

### The haplotype analysis and domestication analysis of *OsCPS1*

**Figure 6:**
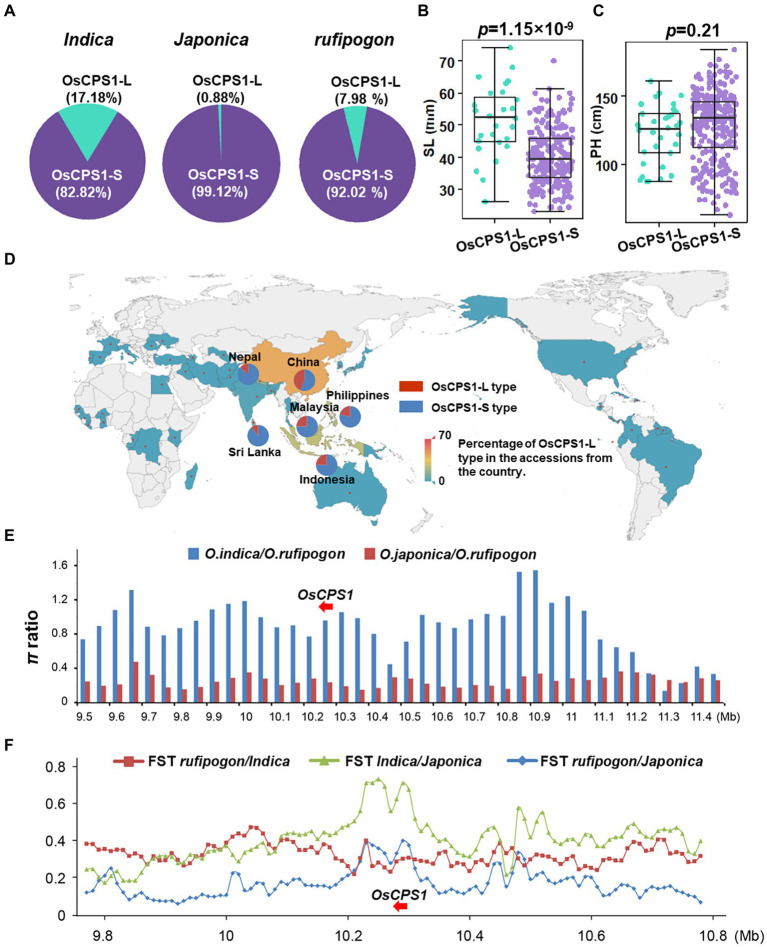
Haplotype analysis and domestication analysis of *OsCPS1*. **(A)** The percentage of 36-bp deletion in *indica*, *japonica* and rufipogon. **(B)** Shoot length of OsCPS1-L type accessions and OsCPS1-S type accessions. **(C)** Plant height of OsCPS1-L type accessions and OsCPS1-S type accessions. **(D)** Geographical distribution analysis of OsCPS1-L type accessions and OsCPS1-S type accessions in the world. **(E)** π (nucleotide diversity) ratio in genome region surrounding *OsCPS1*. Blue bar means π ratio between *indica* and *rufipogon*, red bar means π ratio between *japonica* and *rufipogon.* **(F)** *F_ST_* (fixation index) in the region surrounding *OsCPS1* among rufipogen, *indica* and *japonica.*

To further evaluate the possible function of the 36-bp deletion in the 5' UTR of *OsCPS1*, the *rufipogon* sequencing data was downloaded from NCBI.^2^ All sequencing data of the 302 accessions and *rufipogon* was analyzed. The results identified 2 haplotypes (OsCPS1-L and OsCPS1-S) of *OsCPS1* based on the deletions in the 5′ UTR. In total, 17.18% of *indica* accessions, 0.88% of *japonica* accessions and 7.98% of *rufipogon* accessions harbored the deletion (Figure 6A). We further analyzed the effect of the deletions on shoot length. As shown in Figure 6B, the shoot length differed significantly among the 302 accessions, with the OsCPS1-L type accessions showing longer shoot length (average 53.4 mm) than that OsCPS1-S type accessions (average 45.4 mm; *p* = 1.15 × 10^−9^). Interestingly, the height of mature plants demonstrated no difference between OsCPS1-L and OsCPS1-S accessions (Figure 6C), suggesting that this indel correlates with early seedling vigor, but not with adult plant height. Genealogical networks and geographical distribution of haplotypes were analyzed in a larger international panel contained 509 accessions from 51 countries worldwide. The result showed OsCPS1-S only exists in accessions from a few countries in East or Southeast Asia, including China, Indonesia, Malaysia, Nepal, Philippines and Sri Lanka (Figure 6D).

To identify potential selective signals during domestication or breeding selection of the genome interval surrounding *OsCPS1*, the nucleotide diversity (π) and fixation index (F*st*) were employed as indicators. A significant decrease in nucleotide diversity was observed in the region surrounding *OsCPS1* between *japonica* and *rufipogon*. In contrast, similar diversity was observed between *indica* and *rufipogon* (Figure 6E). In accordance with these results, a significant F*st* peak was identified in the same region between *indica* and *japonica* (Figure 6F). These findings revealed significant genetic divergence in the *OsCPS1* region between *indica* and *japonica*.

## Discussion

Seedling shoot length is an important performance indicator for seedling vigor evaluation, and it is an important agronomic trait in direct seeding of rice. Understanding genetic variations controlling shoot length is valuable for breeding varieties suitable for direct seeding. Here, we used a diverse panel consisting of 302 accessions from 53 countries, presenting phenotypic variance in shoot length (SL) to identify novel QTLs controlling SL.

Six QTLs were found to associate with shoot length and all QTLs were under further investigation. In this study, a QTL located in chromosome 2 (*qSL2*) was studied in detail to find possible causative variations related to shoot length. *OsCPS1*(*LOC_Os02g17780*), which encodes an enzyme for gibberellin (GA) synthesis (Toyomasu et al., 2015), was identified as candidate gene by sequence comparisons and gene expression analysis. *OsCPS1* showed significant expression differences between long and short SL accessions (Figures 3B,C). Furthermore, these expression level differences are strongly correlated with a 36-bp Indel located in the 5' UTR of *OsCPS1*. Assay of the GA content between long and short SL accessions revealed higher expression of *OsCPS1* is associated with increased GA content (Figure 5A). From these results, we hypothesized that *OsCPS1* may be the candidate functional gene underlying *qSL2*, and the 36-bp may be the functional variation leading to the SL phenotype, by modulating the expression of *OsCPS1* and resulting in the differences in GA content.

### *OsCPS1* is a candidate gene for *qSL2*

GA plays an important role in regulating plant growth and development, including seed germination and stem elongation. Bioactive GAs are synthesized from trans-geranylgeranyl diphosphate (GGDP), and GGDP is converted to *ent*-kaurene by CDP synthase (CPS) and *ent*-kaurene synthase (KS; Yamaguchi, 2008). Four *CPS* genes (*OsCPS1*-*4*) have been identified in the rice genome, including a pseudogene (*OsCPS3*; Sakamoto et al., 2004). *OsCPS1* is involved in growth related GA biosynthesis, while *OsCPS2* and *OsCPS4* are involved in the biosynthesis of phytoalexins for defense (Otomo et al., 2004; Prisic et al., 2004; Sakamoto et al., 2004). The *oscps1* mutant exhibited a severely dwarfed phenotype with a decreased level of *ent*- kaurene compared to wild type plants (Sakamoto et al., 2004). Moreover, no bioactive GA_1_ was detected in the *oscps1* mutant (Sakamoto et al., 2004). These data suggested that *OsCPS1* acts as a regulator of GA synthesis. We found that the expression of *OsCPS1* is significant higher in long SL accessions (Figures 3B,C). In addition, the content of GA_1_ and GA_4_ in long SL accessions were higher than that in short SL accessions (Figure 5A). These results suggested that the differences of SL between long and short SL accessions may be due to the difference in OsCPS1-mediated GA content.

Recently, Toyomasu et al. (2015) reported that *OsCPS1* and *OsCPS2* exhibited different tissue-specific expression patterns. The *OsCPS2* transcript level was much lower than that of *OsCPS1* in the basal part of second-leaf sheaths in third-leaf stage rice seedlings. Furthermore, qRT-PCR suggested that *OsCPS1* transcripts mainly localized in vascular bundle tissues, whereas *OsCPS2* transcripts mainly localized in epidermal cells that address environmental stressors. More importantly, *OsCPS2* expression under the *OsCPS1* promoter, but not its native promoter, rescued the *oscps1* mutant dwarfed phenotype, suggesting that tissue-specific expression of *OsCPS* genes is important for regulating growth. Our results showed that, compared with OsCPS1-S type accessions, OsCPS1-L type accessions that harbored a 36-bp deletion in the 5' UTR, showed longer shoot length (Figures 4, 6B), suggesting that the 36-bp deletion may cause differential expression of *OsCPS1* between OsCPS1-S and OsCPS1-L type accessions, leading to the difference in shoot length. Interestingly, the height of mature plants was not different between OsCPS1-L and OsCPS1-S type accessions (Figure 6C), suggesting that this locus may be involved in regulating early seedling vigor, but not adult plant height, which is one of the main factors affecting lodging resistance. Collectively, these data indicated this locus has significant potential in breeding rice varieties suitable for direct seeding.

### *qSL2* may promote shoot growth by OsCPS1-mediated GA signaling in early stage

GA regulates stem elongation by promoting both cell elongation and cell division (Rademacher, 2000). In this study, we identified *OsCPS1*, a gene involved in GA synthesis, as the candidate gene of *qSL2* (Figures 3, 4). Long SL accessions showed an increase in GA_1_ and GA_4_ content (Figure 5A). To answer whether OsCPS1-mediated GA signaling contributes to SL phenotype, RNA-seq was conducted to evaluate gene expression differences between long and short SL accessions.

Chen et al. (2020) reported that photosynthesis, metabolic pathways and biosynthesis of secondary metabolites, and cell wall components were differentially expressed in the sugarcane internodes of the GA-treated plants, indicating their involvement in GA-mediated internode elongation. Consistent with Chen’s report, our results demonstrated genes with higher expression in long SL accessions than those in short SL accessions were significantly enriched for GO terms related photosynthesis (Figure 5B). Several studies have found that GA could increase expression of genes involved in photosynthesis, as well as the photosynthesis rate (Yuan and Xu, 2001; Tian et al., 2016; Chen et al., 2020). We speculated the higher expression of genes associated with photosynthesis may result from higher GA content in long shoot length accessions.

In contrast, genes involved ABA activated signaling pathways showed lower expression in long SL accession than in short SL accessions (Figure 5C). ABA and GA have central and antagonistic roles in regulating rice growth. The antagonistic action between GA and ABA was an important factor regulating the developmental transition from embryogenesis to seed germination (Gómez-Cadenas et al., 2001). However, the crosstalk between GA and ABA, as well as how this crosstalk regulates shoot length needs further investigation. Collectively, the gene expression profile results suggested that *qSL2* may promote shoot growth by OsCPS1-mediated GA signaling at an early stage.

### Haplotype and domestication analysis of *OsCPS1*

Since the 36-bp deletion may be the functional variation underlying *qSL2* conferring SL phenotypes, the origin and evolution of the 36-bp deletion was characterized from wild, *indica* and *japonica* rice. Our results indicated that the 36-bp deletion originated from wild rice and was retained in *indica* accessions (Figure 6A). Surprisingly, the deletion was almost absent from *japonica* accessions, suggesting a strong selective sweep during domestication or/and breeding of *japonica* rice (Figure 6A). Further analysis revealed that the 36-bp deletion only exists in accessions from six Asian countries (Figure 6D), suggesting that this deletion was intentionally selected and retained in these countries. Since this deletion is associated with early seedling growth, we hypothesized that this trait may be an important requirement for rice planting in this region, or for rice environment adaption in this region. In contrast, the lack of this deletion in *japonica* suggested that this phenotype is not suitable for *japonica* or results from a domestication bottleneck.

We determined the nucleotide diversity and fixation index in the genome region surrounding *OsCPS1* among wild rice, *indica* and *japonica* accessions. Nucleotide diversity analysis results revealed a significant decrease in diversity in *japonica* compared to *indica* or wild rice in the region (Figure 6E). However, no similar phenomenon was found in *indica* and wild rice (Figure 6E). F*st* analysis also demonstrated a peak between *indica* and *japonica* in this region (Figure 6F). These results suggested that the region surrounding *OsCPS1* is under strong selection in *japonica*, which is in consistent with the haplotype analysis results. While we provided evidence that *OsCPS1*, and the 5' UTR deletion may be responsible for the SL trait, more detailed investigation on the molecular function of the 36-bp deletion and *OsCPS1* itself is required to fully dissect the process. In future, transgenic methods, including overexpression and CRISPR knockout mutations, could be used to verify *OsCPS1* function.

In summary, by using an international diverse rice panel, we successfully identified germplasm with long shoot length in the early seedling stage of rice growth. This germplasm may be useful as parents for breeding of high seedling vigor varieties. GWAS analysis with deep sequencing data identified novel QTLs controlling SL, and whole genome gene expression profiling by RNA-seq further helped to identify *OsCPS1* as the candidate gene for one of the major QTLs (*qSL2*). These multi-omic data and methods helped to elucidate the genomic variation and molecular basis of modulating shoot length and seedling vigor. A 36-bp deletion in the 5' UTR of *OsCPS1* was characterized as the possible functional variation underlying *qSL2*. The identification of *qSL2* and the candidate gene *OsCPS1* has a potential to accelerate the breeding of superior varieties suitable for direct seeding.

## Data availability statement

The datasets presented in this study can be found in online repositories. The RNAseq data presented in the study are deposited in the NCBI repository (Bioproject: PRJNA839180, Accession number: SRR19259017, SRR19259020, SRR19259023, etc). The DNA sequencing datasets are deposited in the NCBI repository (Bioproject: PRJNA820969, Accession number: SRR19146044, SRR19146039, SRR19146035, etc.) The wild rice sequencing data were downloaded from NCBI (Bioproject: PRJNA657701). The names of the repository/repositories and accession number(s) can be found in the article/Supplementary material.

## Author contributions

YM, JW, DE, SZ, and JZ designed the experiments and wrote and edited the manuscript. YM and JW performed most of experiments and analyzed the data. Other authors assisted in experiments and discussed the results. All authors contributed to the article and approved the submitted version.

## Funding

This work was funded by Guangdong Provincial International Cooperation Project of Science & Technology (2021A0505030048), the Key Areas Research Projects of Guangdong Province (2020B0202090003), Science and Technology Program of Guangzhou (202002030375 and 201804020078), National Natural Science Foundation of China (31901441), Special Fund for Scientific Innovation Strategy-Construction of High Level Academy of Agriculture Science (R2021PY-QF001), the evaluation and operation funds of Guangdong key laboratories (2020B1212060047), the “YouGu” Plan of Rice Research Institute of Guangdong Academy of Agricultural Sciences (2021YG001).

## Conflict of interest

The authors declare that the research was conducted in the absence of any commercial or financial relationships that could be construed as a potential conflict of interest.

## Publisher’s note

All claims expressed in this article are solely those of the authors and do not necessarily represent those of their affiliated organizations, or those of the publisher, the editors and the reviewers. Any product that may be evaluated in this article, or claim that may be made by its manufacturer, is not guaranteed or endorsed by the publisher.

## Footnotes

^1^ http://rice.uga.edu

^2^ www.ncbi.nlm.nih.gov

## Abbreviations

ESV: Early seedling vigor
GWAS: Genome-wide association study
LD: Linkage disequilibrium
MAF: Minor allele frequency
QTLs: Quantitative trait loci
RDP2: Rice Diversity Panel 2
SL: Shoot length
SNP: Single-nucleotide polymorphism
